# Odontogenic Sinusitis: The Selective Role of a Modified Caldwell–Luc Approach in Two CT/CBCT-Documented Cases

**DOI:** 10.3390/diagnostics16162567

**Published:** 2026-08-14

**Authors:** Kamil Nelke, Suat Aktaş, Ömer Uranbey, Marceli Łukaszewski, India Maag, Maciej Janeczek, Agata Małyszek, Michał Gontarz, Klaudiusz Łuczak, Maciej Dobrzyński

**Affiliations:** 1 Maxillo-Facial Surgery Ward, EMC Hospital, Pilczycka 144 Street, 54-144 Wrocław, Poland; klaudiuszluczak@gmail.com; 2 Department of Oral and Maxillofacial Surgery, Faculty of Dentistry, Aydın Adnan Menderes University, Aydın 09100, Türkiye; suat.aktass@gmail.com; 3 Ward of Anaesthesiology and Intensive Care, Vratislavia Medica Hospital, 51-134 Wrocław, Poland; marceliluk@gmail.com; 4 Private Dental Office, Pawia 56 Street, 54-144 Wrocław, Poland; indiamaag@gmail.com; 5 Department of Biostructure and Animal Physiology, Wrocław University of Environmental and Life Sciences, Cypriana K. Norwida 31 Street, 50-375 Wrocław, Poland; maciej.janeczek@upwr.edu.pl (M.J.); agata.malyszek@upwr.edu.pl (A.M.); 6 Department of Cranio-Maxillo-Facial Surgery, Maxillo-Facial Surgery Clinic, University Hospital in Cracow, Macieja Jakubowskiego 2 Street (Nowy Prokocim), 30-688 Krakow, Poland; michal.gontarz@uj.edu.pl; 7 Department of Pediatric Dentistry and Preclinical Dentistry, Wrocław Medical University, Krakowska 26, 50-425 Wrocław, Poland; maciej.dobrzynski@umw.edu.pl

**Keywords:** odontogenic sinusitis, tooth-bearing structures, maxillary sinus, Caldwell–Luc operation, intranasal surgical procedures

## Abstract

Tooth-bearing structures are commonly involved in the spread of various odontogenic lesions. Radicular and dentigerous cysts are among the most common odontogenic cysts, exhibiting significant variations in size and morphology within the jawbones. On the other hand, the influence of dental caries, periodontal diseases, and periapical lesions extends beyond tooth-bearing bone and also affects adjacent anatomical structures, especially the maxillary sinuses. In addition to untreated odontogenic lesions, treatment-related complications in the posterior maxilla may also disturb the sinus environment, particularly when oroantral communication is not recognized or adequately managed. The etiology of sinusitis is multifactorial; however, tooth-related infections are the primary cause of odontogenic sinusitis (ODS). Routine upper posterior tooth extraction may also become clinically significant when pre-existing sinus mucosal changes, close sinus floor proximity, or persistent oroantral communication are overlooked. When ODS is undiagnosed or overlooked on a classic computed tomography or a cone beam computed tomography (CBCT) scan, it is frequently inadequately managed by otolaryngological interventions alone; this leaves the underlying dental pathology unaddressed. Consequently, the utilization of CT/CBCT and close collaboration between dentists, oral surgeons, and otolaryngologists are essential for the successful management of these conditions. In advanced cases of ODS, isolated intranasal otolaryngological procedures are often insufficient. Comprehensive management relies on the elimination of all sources of odontogenic infection through endodontic therapy, dental extractions, and localized bone curettage. While the traditional Caldwell–Luc approach (CLA) is not commonly used nowadays, it remains an important surgical option in select cases, especially when infection spreads beyond tooth-bearing structures or soft tissue abscesses are present. This paper presents the radiological and clinical findings of two ODS cases with different etiologies and demonstrates how these findings influenced surgical planning. The aim is not to introduce the Caldwell–Luc approach as a novel technique but to illustrate its selective role when the direct treatment of the odontogenic source and oroantral defect is required.

**Figure 1:**
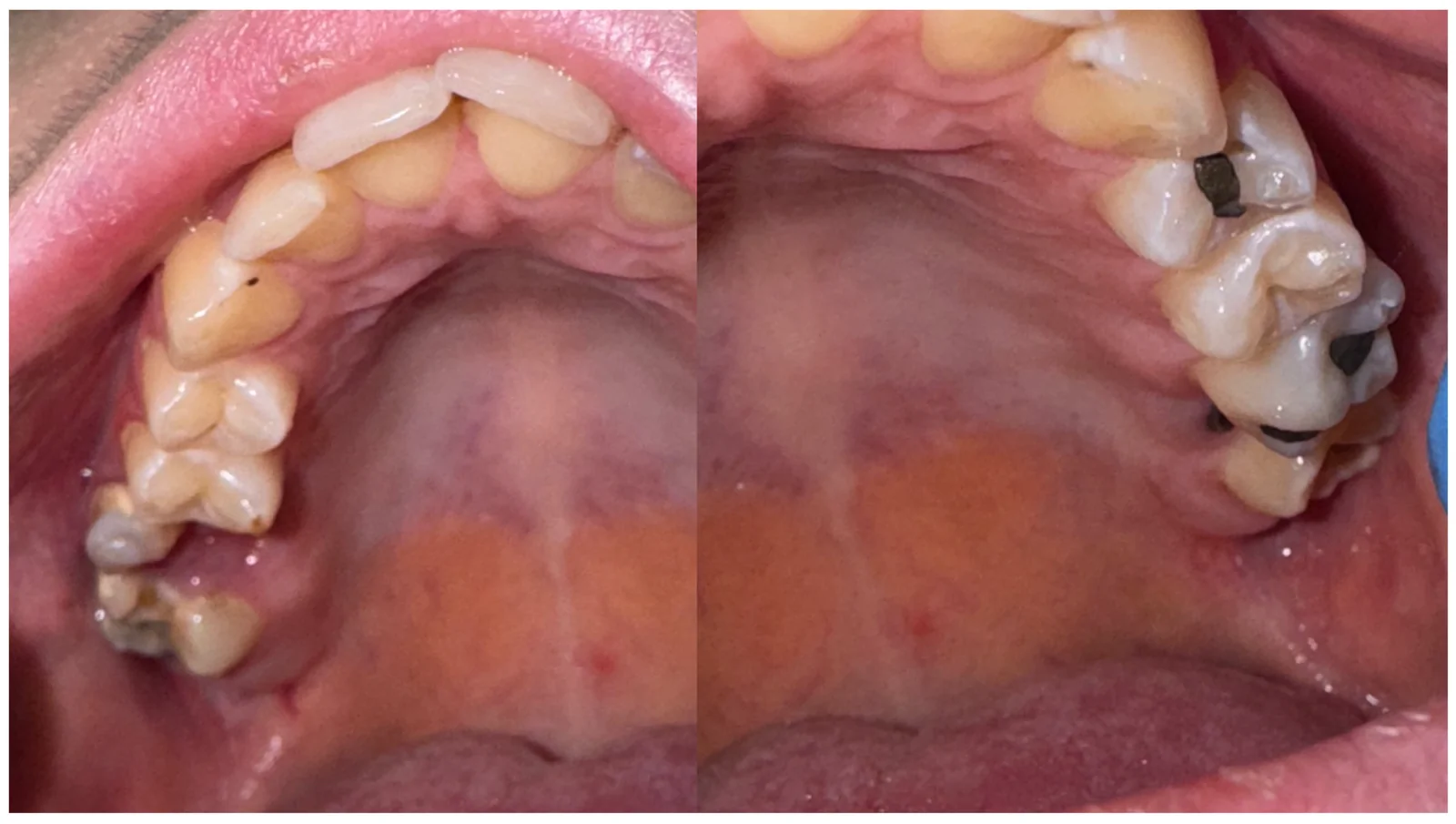
In many instances, clinical findings from intraoral examinations of teeth and tooth-bearing structures do not correlate with cone beam computed tomography (CBCT) images. Local dental factors can influence the severity of gingival inflammation, the development of periapical lesions, and the extent of dental caries, thereby altering a patient’s symptomatology over time or even resulting in completely asymptomatic presentations. In the maxilla, the close proximity of the alveolar recess of the maxillary sinus to the tooth-bearing structures, along with the limited thickness of the bone between the inferior sinus wall and the root apices, facilitates the rapid spread of odontogenic infections. The presence of gingival fistulas, bone asymmetry, localized swelling, or oral mucosal inflammation, as well as active abscess formation, strongly suggests a local odontogenic etiology [1,2,3,4,5,6,7,8]. Patients frequently seek otolaryngological consultation because of nasal symptoms, while the underlying dental origin may remain underestimated. In the presented 25-year-old female, a right-sided buccal abscess initially responded to drainage and antibiotic therapy; however, the underlying periapical pathology was not sufficiently recognized. Following persistent nasal discharge, sinus pain, and impaired nasal patency, computed tomography (CT) imaging of the sinuses was scheduled. Quite often, the poor sinus condition does not fully correlate with visible oral cavity symptoms, which is why an evaluation of surrounding tooth-bearing structures is crucial [9,10,11,12,13,14,15,16,17]. Nowadays, endoscopic intranasal approaches (ESS/FESS—endoscopic sinus surgery/functional endoscopic sinus surgery) are quite popular and used by otolaryngologists. The necessity of approaches for the oronasal and/or oroantral fistula requires its direct excision in an intraoral approach and coverage with one of the used flap techniques to ensure its closure, revision of the maxillary sinus, and drainage, which can be easily achieved via oral cavity access. Although the Caldwell–Luc approach and its modifications are already well described, a limited intraoral approach may still provide useful direct access to the tooth-bearing maxilla in selected cases requiring the removal of the odontogenic pathology or closure of an oroantral defect [1,2,3,4,5,6,7,8,9].

**Figure 2:**
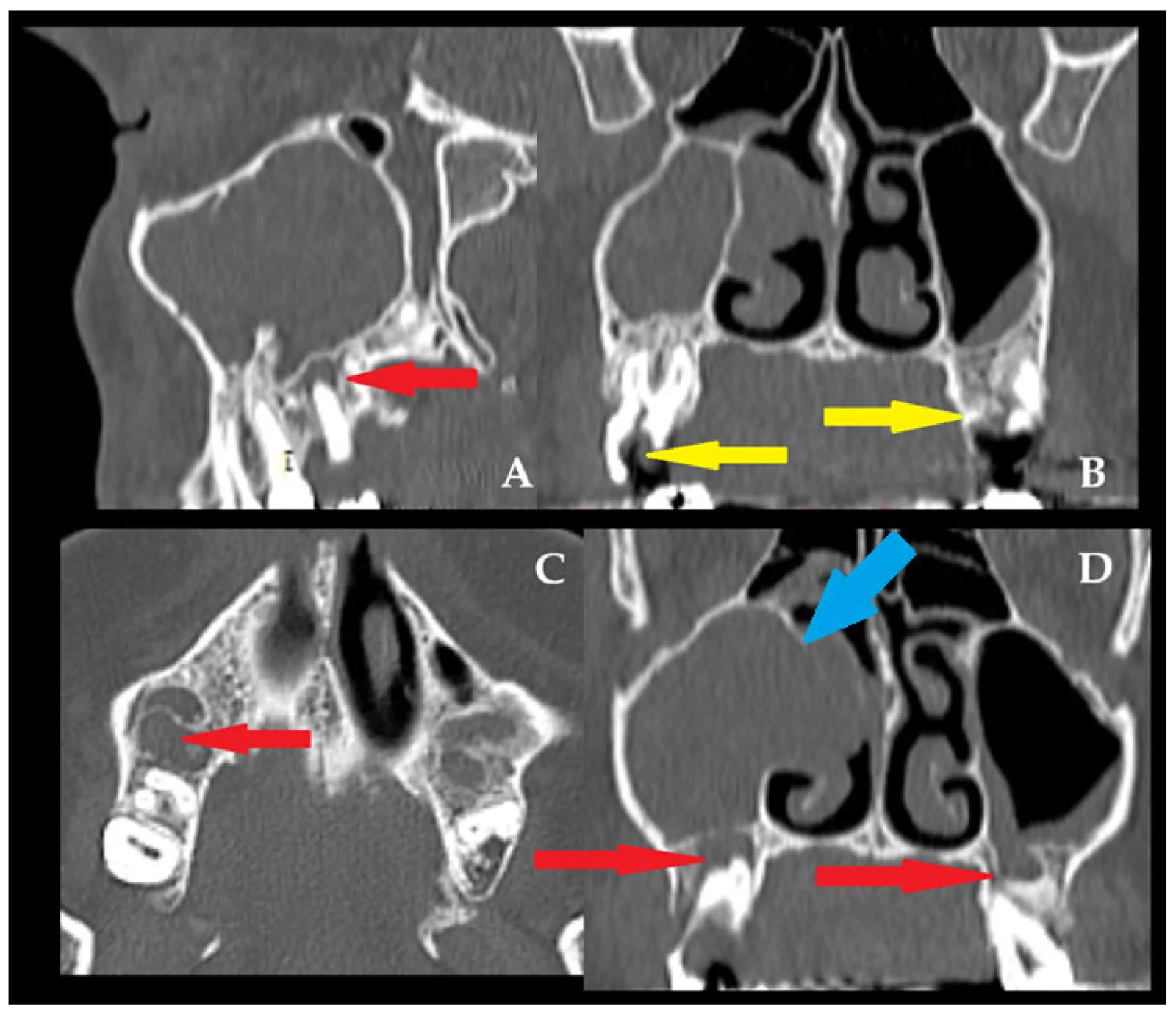
It might seem unusual, however, sometimes the correlation between dental-related radiolucent areas in the tooth-bearing structures might be easily misdiagnosed because of the variation in the alveolar recess depth, presence of Underwood septa, some anatomical variations in the bone shape of the maxillary sinus, or the condition of the underlying bone [8,9,10,11,12,13,14,15]. The so-called odontogenic sinusitis (ODS) is quite often neglected, underrated, and frequently ignored until some other obvious improved and significant tooth-related factors are present. It is also worth remembering that small lesions with uncertain behavior in the tooth periapical or periodontal area might cause ODS appearances in various stages. The scheduled CT revealed a complete lack of ventilation of the right maxillary sinus, followed by total opacity, with fluid-like and polyp mass formation extending towards the nasal cavity in between the inferior and middle conchae (blue arrow) [15,16,17,18,19,20,21]. In Figure 2, the space under the lower conchae and the nasal septum remains partially blocked and covered by the nasal polyps and some sinus secretion. Red arrows (**A**,**C**,**D**) show active periapical lesions with cystic formation because of advanced dental caries ((**B**) yellow arrow). Adjacent to the mentioned arrows, a radiolucent round area representing periapical lesions is clearly visible. Radiologically, ODS is suggested by unilateral maxillary sinus opacification or focal mucosal thickening adjacent to a diseased tooth, particularly when associated with a periapical or periodontal lesion, oroantral communication, a foreign body, or focal sinus floor discontinuity. In contrast, rhinogenic sinusitis more commonly presents with bilateral or multisinus mucosal disease centered on the OMC, without a direct dental or sinus floor abnormality [12,13,14,15,16,17,18]. It is quite important to notice that the left maxillary sinus remains only partially affected by the ODS because increased sinus mucous membrane thickening was visible (**B**,**D**). The evaluation of the osteomeatal complex (OMC), nasal obstruction, and possible ethmoidal involvement remains important for surgical planning and for deciding between ESS/FESS and combined approaches [4,5,6,7,8,9,10,11,12]. In the present conventional sinus CT images, the tooth-bearing regions were clearly visualized, allowing active ODS to be readily identified. However, ESS/FESS provides limited access to the alveolar recess and does not directly address teeth, odontogenic cysts, periapical or osseous lesions, or oroantral and oronasal communication. It also does not permit direct ostectomy, bone revision, or the closure of associated fistulas. Therefore, selected cases requiring simultaneous treatment of the odontogenic source and maxillary sinus may benefit from an intraoral or combined intraoral–endoscopic approach, according to the extent and location of the disease.

**Figure 3:**
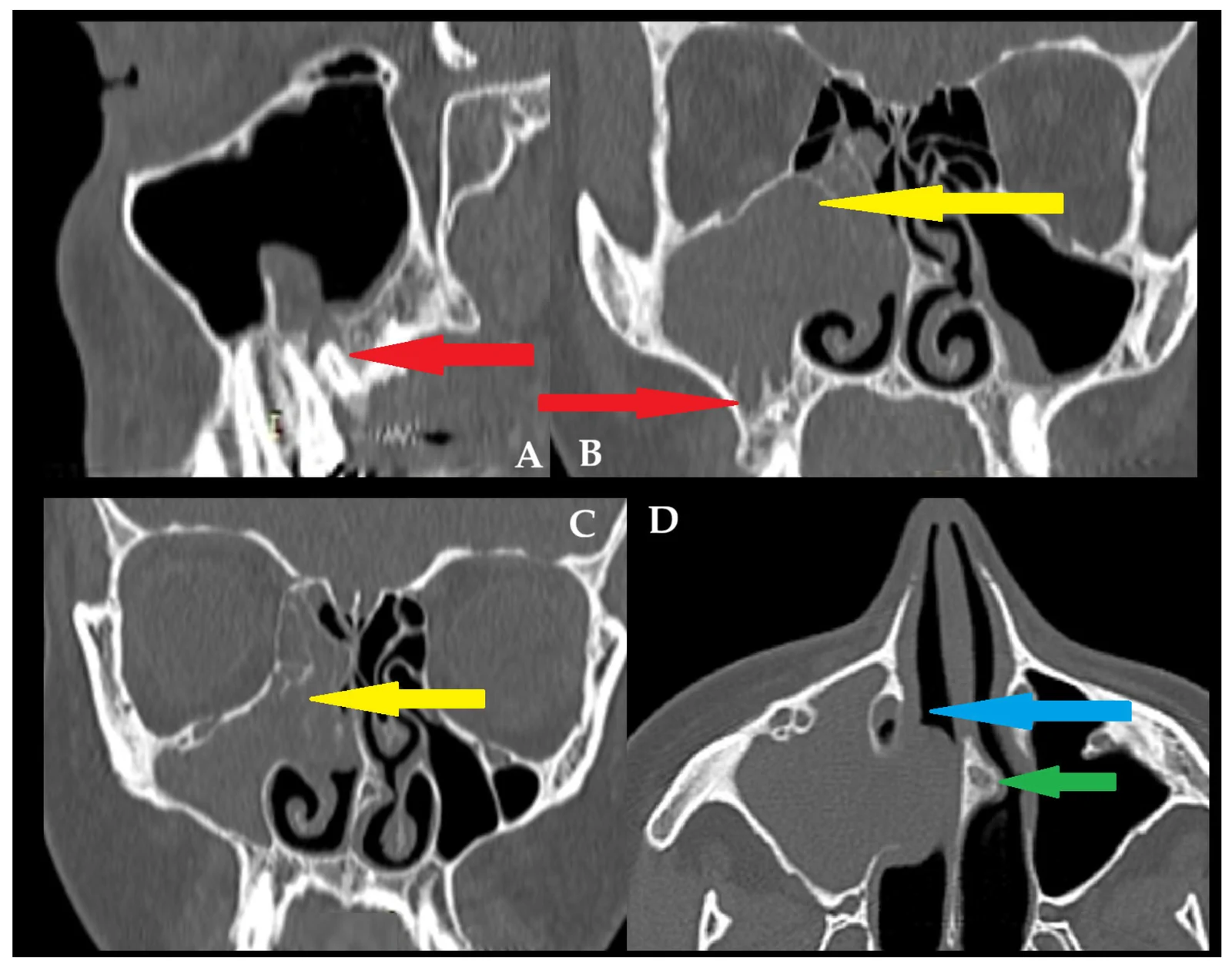
The key surgical and radiological remarks should consider the scope of nasal patency ((**B**), yellow arrow), cortical bone condition (thickening, loss of bone, asymmetry) ((**A**,**B**), red arrow), sinus volume (hypo-/hyper-ventilation, possible SSS—silent sinus-related), possible extra-sinus spread to soft tissues, tooth-bearing structure condition (periapical lesion, cyst, periodontitis, etc.), and, most importantly, the condition of the osteal meatal complex ((**C**), yellow arrow) [8,9,10,11,12,13,14,15,16]. The importance of a detailed preoperative CT/CBCT anatomical assessment has also been emphasized for advanced sinonasal procedures [17,18,19,20,21]. From a clinical perspective, the urgency of sinus drainage and the removal of active worrisome inflammation factors might reduce the inflammation/infection spread towards the eye socket, nasal cavity, ethmoids, and the buccal areas ((**D**), blue arrow). In cases of further decreased OMC ventilation and a severely deviated nasal septum with some spur or cartilage abnormalities, an additional septoplasty could be considered ((**D**), green arrow) for nasal ventilation improvement. In cases where ESS/FESS, laryngological intervention, and dental surgeon/dental appointments might be troublesome to achieve in time, it is also possible to perform a one-stage surgery based on the Caldwell–Luc approach (CLA) combined with sinus drainage, OMC irrigation and the removal of all dental/tooth-related sources of inflammation (dental roots, periapical lesions or other) and evaluate gathered specimens in microbiological–histopathological studies and ensure that there is a reduction in the troublesome clinically active source of inflammation. This approach serves as an effective primary intervention, preparing the region for subsequent surgical, otolaryngological, and dental treatments. Based on the image of the ODS in Figure 3, the patient was scheduled for an intervention under general anesthesia, including anterior small window antrotomy via the modified CLA with sinus drainage, bone ostectomy, local curettage, periapical lesion with tooth removal, and stable oroantral communication closure with Bichat fat pads. An additional step consisted of the drainage, irrigation, and curettage of the osteomeatal complex from the intraoral CLA with additional surgical fistula formation via the lateral nasal bone wall towards the area under the inferior conchae for additional Foley catheter drainage for 24/48 h (irrigated with saline three times per day). The surgical procedure was uneventful. Postoperatively, the patient continued intravenous antibiotic therapy consisting of Taromentin (3 × 1.2 g, Polfa Tarchomin, Warszawa, Poland) and Metronidazole (3 × 500 mg, Polpharma, Starogard Gdański, Poland). The patient was discharged on the first postoperative day with a prescription to continue oral medication for an additional 10 days. Postoperative oral hygiene was maintained by rinsing the oral cavity three times daily with a 0.1% chlorhexidine (CHX) solution, diluted with mineral water at a 1:7 ratio. Additionally, Elugel (Pierre Fabre Oral Care, Paris, France) was prescribed for topical application to the surgical wound. Since the sinus was not purulent and two-day sinus irrigation with saline led to the patient’s better overall local condition, additional nasal steroid drops (Dymista, nasal corticosteroid, MEDA Pharma Bad Homburg, Hessen, Germany—two times per day) with irrigation nasal rinses (Sinus-Rinse, NBTY Manufacturing INC, New York, NY, USA—4 times per day) were prescribed. Because of some slight numbness of the upper lip, Nurovit (320 mg twice daily) was additionally prescribed. Each tablet contains 100 mg of vitamin B1, 200 mg of vitamin B6, and 0.2 mg of vitamin B12 (G.L. Pharma GmbH, Gerot Lannach, Vienna, Austria). The patient was advised to follow up with a daily oral and nasal hygiene regimen with details. The following limitations of the CLA include: possible damage to the infraorbital nerve (a branch from the trigeminal nerve); loss of bone related to the scope, shape and size of the antrostomy window; possible damage to adjacent teeth, apices and periapical tissues of adjacent teeth; possible damage to healthy mucosa of the maxillary sinuses; damage to the Stenon duct; possible bone loss of tooth-bearing structures and the alveolar recess of the maxillary bones; formation of fistula; flap necrosis; or insufficient coverage of the oroantral/oronasal fistulas requiring additional surgery [12,13,14,15,16,17,18,19,20,21,22,23,24,25]. These risks, including late postoperative maxillary cyst formation from an entrapped respiratory epithelium, may be minimized by limited and accurately positioned antrostomy, the preservation of the infraorbital nerve and healthy mucosa, the avoidance of mucosal entrapment, tension-free closure, and long-term follow-up [25,26,27,28,29,30,31,32].

**Figure 4:**
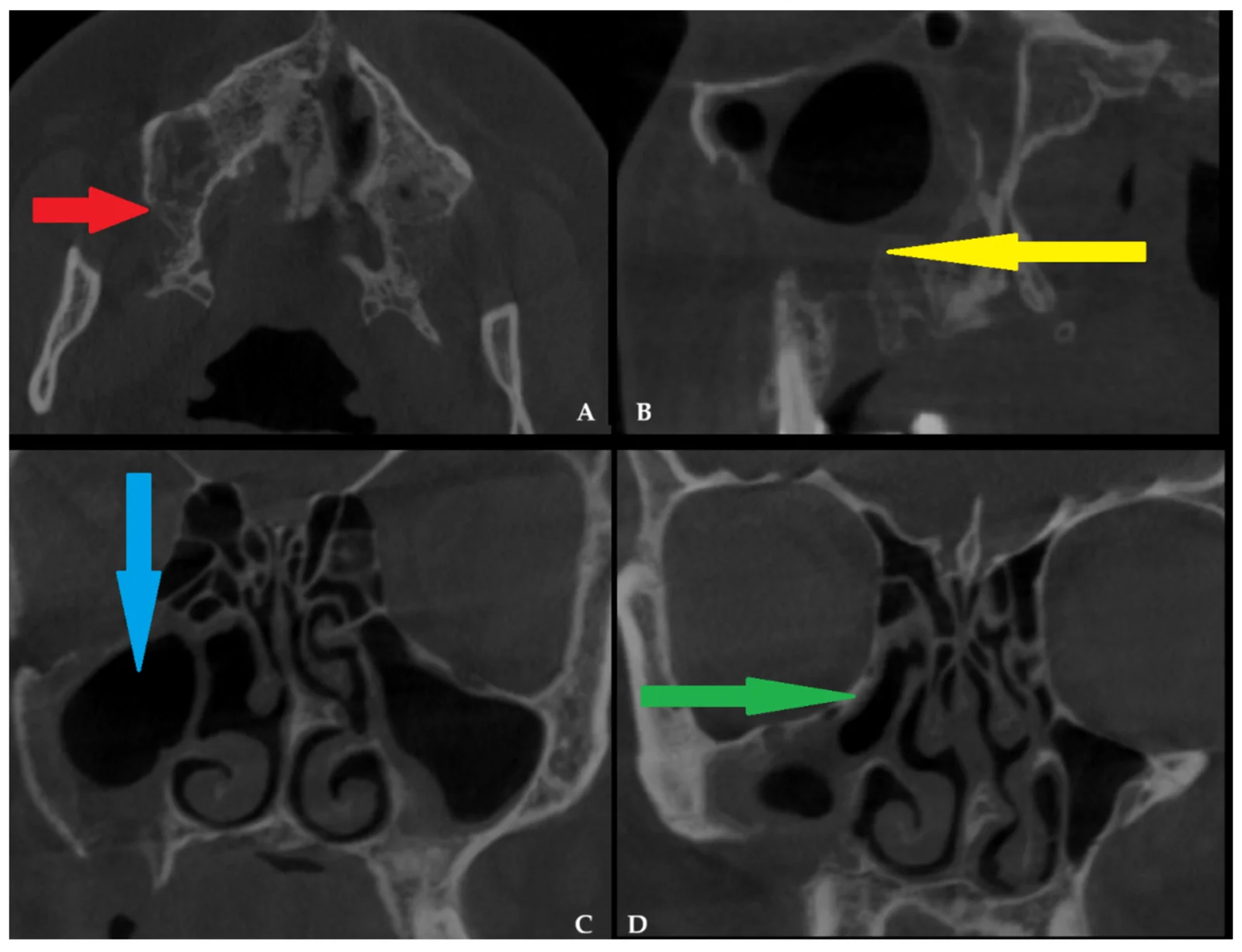
Early postoperative results at 3 months revealed very good and satisfying results visible in a CBCT (cone beam computed tomography) study (20 × 20 FOV (field of view); imaging protocol based on RayScan S 5471.3 mGy, RayCompany Ltd., Samsung 1-ro, Hwaseong-si, Gyeonggi-do, Republic of Korea). Although traditional and less frequently utilized today, the CLA remains highly valuable for managing the maxillary sinus. When combined with simultaneous tooth extraction, the complete curettage/removal of periapical lesions, and bone ostectomy ((**A**,**B**), red and yellow arrows), this approach facilitates thorough sinus irrigation ((**C**), blue arrow). Furthermore, establishing dual sinus drainage consisting of OMC irrigation with curettage ((**D**), green arrow) and an inferior nasal antrostomy not only restores sinus ventilation but also eliminates progressive inflammatory processes, effectively halting the recurrence and spread of ODS. Such an approach should take into consideration the presence of active tooth-related periapical lesions, their further possible spread into the sinus, and a few episodes of ODS exacerbation [19,20,21,22,23,24,25]. On the other hand, the timing of this procedure should consider the time without any active soft tissue inflammation/purulent formation and the simultaneous combination of all of the mentioned bone, tooth, sinus, and nasal procedures to improve the patient’s condition and prepare the patient for any typical ODS treatment with FESS/ESS and/or dental treatment when necessary. This early satisfactory outcome was achieved when the simultaneous pharmacological therapy was intense in both pre- and postsurgical periods, while the single surgical approaches focused on removing teeth and dental- and sinus-related factors at once [25,26,27,28,29,30]. In this case, the otolaryngologist, based on worrisome nasal and sinus problems, improved the diagnostic accuracy with CT to fully visualize the scope of sinonasal problems. Furthermore, the intraoral examination revealed fully healed flaps with no evidence of an oroantral fistula, while the endoscopic evaluation showed no purulent discharge within the nasal cavity. The patient reported improved nasal breathing, reduced nasal fetor, and the complete resolution of sinus pain. The 3-month CBCT showed stable sinus aeration without recurrent opacification, while endoscopic examination showed no purulent discharge; no clinical recurrence was observed at 12 months.

**Figure 5:**
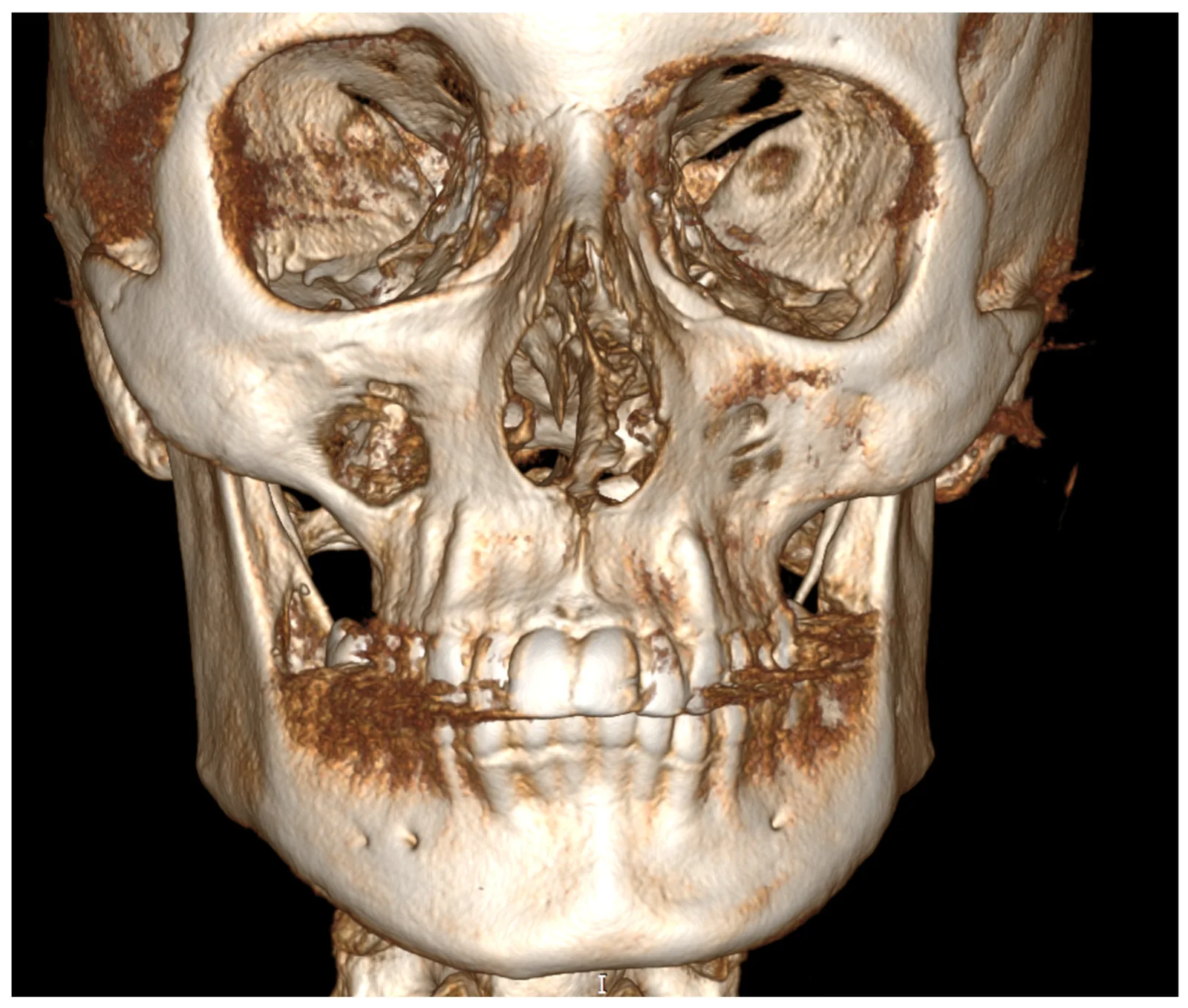
The CLA requires preparation of an antrotomy window whose size and position should be planned according to the extent of access, the periapical pathology, the cyst location, and possible oroantral communication closure. The superior aspect of the approach should take into consideration the infraorbital foramen and its nerve, while the inferior aspect should consider the tooth apices. The 3D-CBCT figure presents the bone loss in the right maxillary sinus wall, due to the CLA, without any significant loss of bone shape, volume, or function. Nevertheless, the CLA is not very common nowadays because of ESS/FESS and other endoscopic approaches. Although it is no longer a routine first-line approach, the Caldwell–Luc procedure may remain a selective surgical option when direct access to the alveolar recess, tooth-bearing structures, or a persistent oroantral defect is required [5,6,7,8,9,10,11,12,17,18,19,20,21,22,28,29,30]. In general, ESS/FESS is preferred when the main problem is osteomeatal complex obstruction or broader sinonasal involvement, whereas a limited Caldwell–Luc approach may be considered for the direct removal of an alveolar recess pathology or foreign bodies and for the simultaneous closure of an oroantral defect; both approaches can be combined when these indications coexist [22,27,28,29,30,33]. A simplified treatment algorithm comparing conservative dental treatment, ESS, the modified Caldwell–Luc approach, and combined approaches is provided in Supplementary Figure S1 [22,27,28,29,30,33]. Perhaps the necessary bone drilling, loss of part of the sinus anterior wall, the down-regulation of maxillary ciliary mucous flow, and sinus ventilation could be some arguments against this approach, yet still, when the source of dental-related infection/inflammation and ODS is caused by still-present dentition and tooth–bone-bearing structures are present, then the CLA should be carefully considered. Some authors emphasize the role of ESS/FESS, others the CLA, while some tend to combine FESS + CLA and perform a dual, two-approach intraoral–intranasal combined treatment, which should also be considered in some special cases. During the patient’s clinical examination, the outcomes just after the procedure and 12 months were stable. Firstly, the considered septoplasty with FESS/ESS was moved to a later stage as a precaution for any ODS relapse or its formation in the opposite left-sided maxillary sinus.

**Figure 6:**
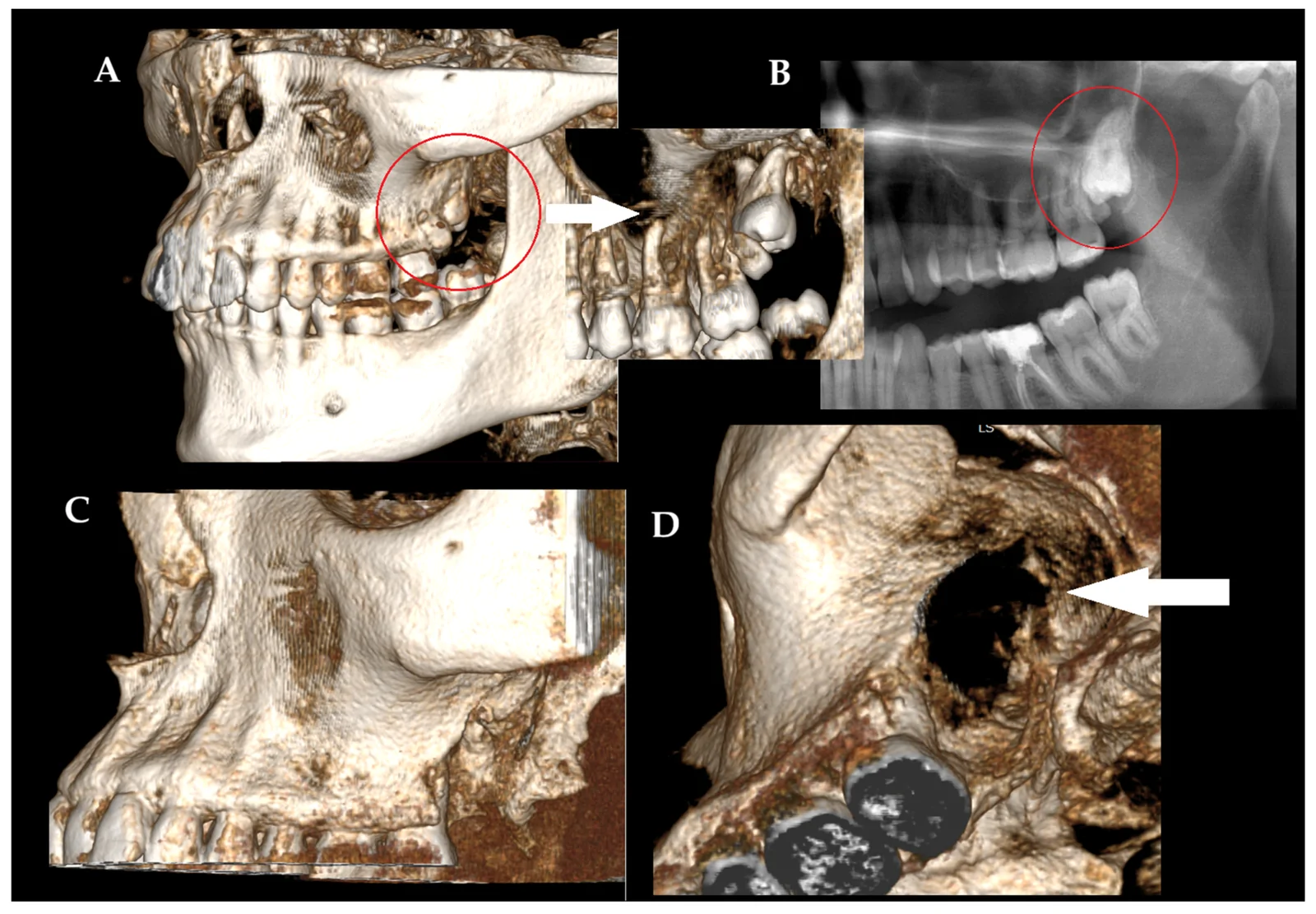
It is important to emphasize that ODS may arise from both odontogenic factors and surgeon- or patient-related factors. This may occur when oroantral communication is not identified promptly or when a small, initially inconspicuous pathway enlarges because postoperative instructions are not adequately followed. Such iatrogenic complications may also lead to ODS, although they represent a distinct clinical pathway. Nevertheless, the main treatment principles remain similar and focus on eliminating the odontogenic source, draining the sinus, and achieving the secure closure of the oroantral defect through a carefully planned intraoral approach. In selected cases, the Caldwell–Luc approach may be required to provide adequate sinus access and reliable closure. The etiology of ODS can have a variety of factors. What appears to be a simple extraction in the posterior maxilla may in reality be the beginning of a sinus problem whose true mechanism is revealed only after the operation, because in this region the danger lies not only in the removal of the tooth, but in the biological and surgical consequences of postoperative communication between the oral cavity and the maxillary sinus. Odontogenic sinusitis may not only arise from an active dental infection but may also be triggered or worsened when a pre-existing sinus vulnerability meets inadequately controlled oroantral communication after extraction, particularly in upper posterior teeth with close sinus proximity [28,29]. For the second case, CBCT images were acquired using a Planmeca (Helsinki, Finland) ProMax 3D Mid unit with a 16.0 × 6.2 cm FOV, 0.4 mm isotropic voxel size and reconstructed slice thickness, 400 × 400 matrix, 155 slices, and exposure settings of 90 kVp and 8 mA; axial, coronal, and sagittal multiplanar reconstructions were evaluated. In the present CBCT images, a 22-year-old female who was initially considered as a routine asymptomatic wisdom tooth case had occasional chronic sinusitis in her history, although this was not emphasized in the initial anamnesis and did not influence the original surgical risk perception. After the extraction of the maxillary third molar together with an additional distomolar, spontaneous drainage from the socket, halitosis, pain on palpation, and discomfort in the extraction area and ipsilateral midface gradually revealed that the postoperative oroantral relationship had become clinically relevant. Medical treatment with amoxicillin/clavulanic acid (AUGMENTIN-BID 1000 mg tablet, 2 × 1 tablet/day; GlaxoSmithKline, İstanbul, Türkiye), ornidazole (BİTERAL 500 mg tablet, 2 × 500 mg/day; DEVA, Tekirdağ, Türkiye), and decongestive therapy (KONGEST 300 mg/2 mg/5 mg tablet, 1 tablet/day, Sanofi, İstanbul, Türkiye) was insufficient, and the region was therefore reopened and drained through a posteriorly positioned CLA-type access point; the sinus was curetted up to the ostium, irrigated with saline, additionally washed with rifampicin diluted in saline, and then closed primarily in layers with the support of Bichat’s buccal fat pad, which was followed by advice to avoid barotrauma, after which the patient became asymptomatic and the culture remained non-specific, supporting the continuation of empirical antibiotic therapy. (**A**), preoperative CBCT/tomographic review image showing the impacted maxillary third molar and distomolar, marked with a red circle, emphasizing that in such surgery, the feared scenario is not only accidental displacement of teeth or roots into the sinus but also failure to anticipate and manage a potentially large postoperative oroantral defect. (**B**), panoramic radiograph of the same region, demonstrating preoperative mucosal thickening and partial sinus opacity, a finding that may appear radiologically modest yet becomes highly relevant when extraction further destabilizes sinus homeostasis. (**C**,**D**), three-month postoperative views of the posterior CLA defect, indicated by the white arrow, illustrating a practical advantage of this posteriorly oriented access in selected cases: direct treatment of the diseased sinus compartment through the same surgical field, easier closure with Bichat-based layered repair, and avoidance of the creation of a second larger anterior bony window, although the choice of approach must always be individualized according to the defect size, the sinus burden, and the extent of the local infection [30,31].

**Figure 7:**
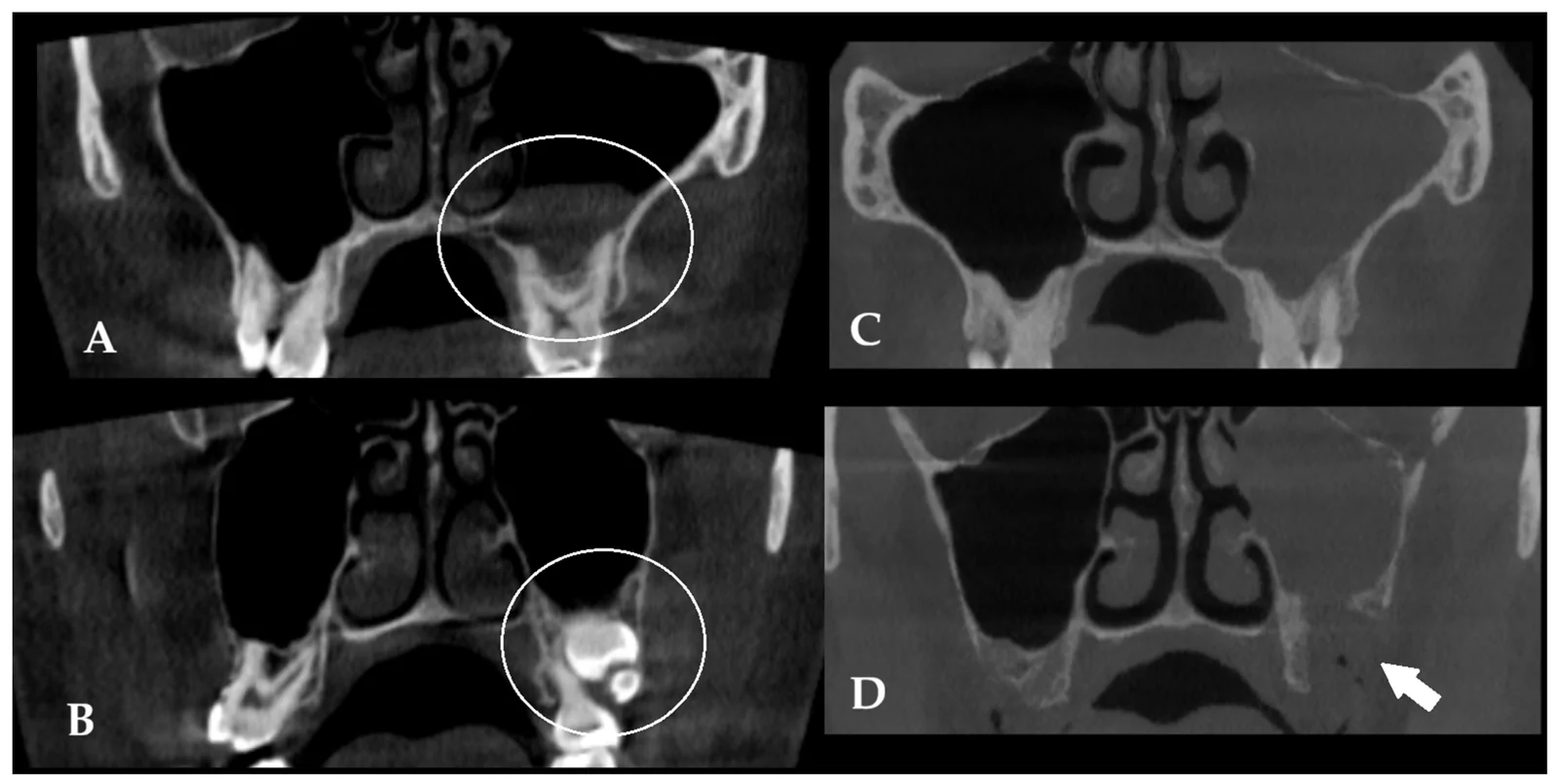
Preoperative imaging may reveal only chronic sinus thickening, yet overlooked, underestimated, or inadequately sealed oroantral communication can trigger dramatic, near-complete opacification shortly after surgery. This rapid radiological transformation provides a striking visual demonstration of how acutely a chronic sinus condition can exacerbate following routine maxillary posterior tooth extraction [32]. The following CBCT sequence captures this progression over weeks and underlines that the preoperative CBCT evaluation should never be limited to the position of the tooth alone. It should also include a careful assessment of the sinus mucosal status, the continuity and thickness of the sinus floor, the relationship of impacted or supernumerary teeth to the alveolar recess, the possibility of creating a large defect, and the presence of pre-existing changes that may reduce postoperative tolerance [34,35]. (**A**,**B**), preoperative coronal CBCT sections before routine third molar surgery showing chronic mucosal thickening together with the maxillary third molar and distomolar, marked with white circles, indicating that even before the intervention, the sinus was not entirely healthy. (**C**,**D**), postoperative coronal CBCT sections obtained one month later, where the white arrow indicates the persistent oroantral relationship that was not adequately managed during the early postoperative period, while the marked increase in sinus opacity demonstrates the rapid evolution of more severe odontogenic sinusitis. In such situations, the literature repeatedly stresses that proper postoperative management begins intraoperatively, with a careful inspection of the socket, cautious use of the Valsalva maneuver where appropriate, and recognition that even seemingly small communication may become clinically significant when accompanied by sinus disease [36,37]. Depending on the defect size and infection status, management options may include socket protection with absorbable hemostatic or gelatin sponge materials such as Spongostan-type products, collagen support (BIOPAD, Euroresearch, Milan, Italy), figure-of-eight sutures, sinus precautions, decongestants, empirical antibiotics when indicated, and close short-term follow-up, whereas persistent, larger, infected, or epithelialized defects may require formal closure with a buccal advancement flap, a palatal flap, Bichat’s buccal fat pad, or combined intraoral and sinus surgery, including endoscopic or CLA-based management in selected advanced cases [37,38]. Bichat’s fat pad is particularly useful for moderate-sized posterior oroantral defects, compromised local mucosa, or failed local flaps because of its rich vascularity and easy mobilization. However, its limitations include a restricted reach for anterior or very large defects, inability to enable bony reconstruction, limited availability after previous use, and possible partial necrosis or dehiscence [25,28,31,37]. These images, therefore, emphasize that the postoperative phase is not merely a period of healing observation but a decisive biological window in which a failure to diagnose and manage the oroantral relationship may convert a routine extraction into a surgically relevant odontogenic sinusitis scenario. Comparative studies suggest that CBCT offers high spatial resolution for dentoalveolar bone and may demonstrate better cortical defects or tooth displacement, whereas conventional multidetector CT provides broader sinonasal coverage and better soft tissue assessment; therefore, the two modalities should be considered complementary rather than one being universally superior [39,40,41,42,43]. In the first case, the extensive periapical and cystic pathology required direct tooth removal. In the second case, persistent posterior oroantral communication required direct sinus curettage and simultaneous closure with Bichat’s fat pad; therefore, an exclusive endoscopic approach was considered insufficient in both cases. These interesting images emphasize that the Caldwell–Luc approach should not replace ESS routinely but may remain useful in carefully selected ODS cases when direct intraoral access is clinically required.

## Data Availability

The datasets used and/or analyzed during the current study are available from the corresponding author upon reasonable request.

## Supplementary Materials

The following supporting information can be downloaded at: https://www.mdpi.com/article/10.3390/diagnostics16162567/s1, Figure S1: Simplified treatment algorithm for odontogenic sinusitis.
