# Supplementary material for: Odontogenic Sinusitis: The Selective Role of a Modified Caldwell–Luc Approach in Two CT/CBCT-Documented Cases
Source: Diagnostics (Basel). 2026 Aug 14;16(16):2567. doi: 10.3390/diagnostics16162567 (PMC13512402; doi:10.3390/diagnostics16162567)
Supplement: Supplementary file 1 [file diagnostics-16-02567-s001.zip › diagnostics-4466551-supplementary.pdf]

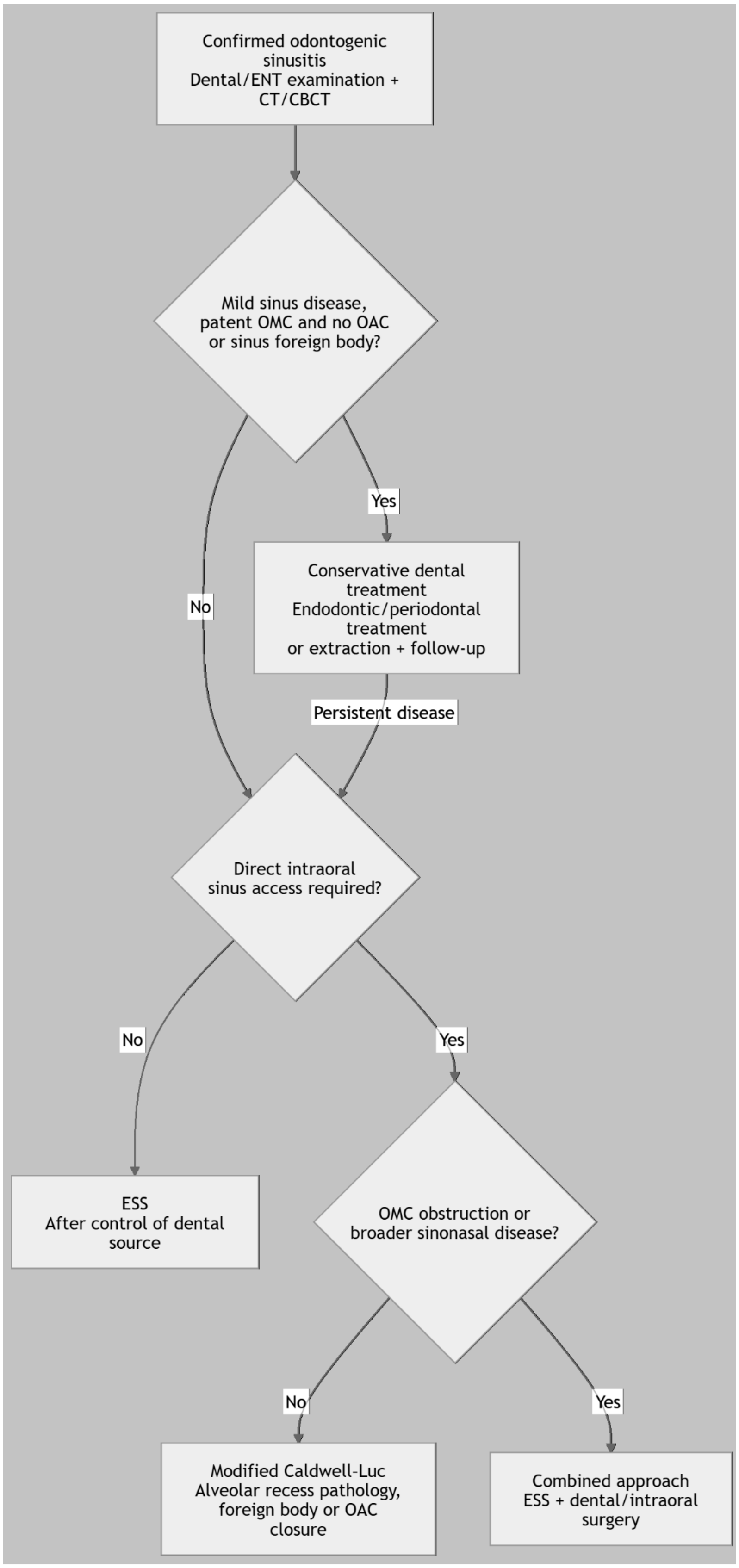

Figure S1. Simplified treatment algorithm for odontogenic sinusitis. Treatment selection is based on the extent of sinonasal disease, OMC involvement, control of the odontogenic source, and the requirement for direct intraoral access. OMC, osteomeatal complex; OAC, oroantral communication; ESS, endoscopic sinus surgery.
